# Perspectives on long-acting injectable HIV antiretroviral therapy at an alternative care site: a qualitative study of people with HIV experiencing substance use and/or housing instability

**DOI:** 10.1186/s12954-023-00730-z

**Published:** 2023-01-10

**Authors:** Laura Fletcher, Shana A. B. Burrowes, Ghulam Karim Khan, Lora Sabin, Samantha Johnson, Simeon D. Kimmel, Glorimar Ruiz-Mercado, Cassandra Pierre, Mari-Lynn Drainoni

**Affiliations:** 1 Section of Infectious Diseases, Boston Medical Center, 801 Massachusetts Avenue, Crosstown Building, 2nd Floor, Room 2015A, Boston, MA 02118 USA; 2 Section of Infectious Diseases, Department of Medicine, Boston University Aram V. Chobanian & Edward Avedisian School of Medicine, Boston, USA; 3 Section of General Internal Medicine, Boston Medical Center, Boston, USA; 4 Department of Global Health, Boston University School of Public Health, Boston, USA; 5 Section of Infectious Diseases, Section of General Internal Medicine, Boston Medical Center, Boston University Aram V. Chobanian & Edward Avedisian School of Medicine, Boston, USA; 6 Department of Health Law Policy and Management, Boston University School of Public Health, Boston, USA

**Keywords:** HIV, Long-acting injectable ART, Adherence, Qualitative, Care delivery models, Substance use, Homelessness

## Abstract

**Introduction:**

Adherence to daily oral antiretroviral therapy (ART) and regular clinic appointments can be challenging for individuals who experience adverse social determinants of health. Long-acting injectable ART administered outside of traditional clinic settings may be a promising solution to adherence barriers, but additional research is needed to assess patients’ perspectives. This study assessed perspectives of people living with HIV (PLWH) who had difficulty with adherence to traditional HIV care models and evaluated feasibility and acceptability of receiving a long-acting ART injection at a location outside of a traditional HIV clinic to address barriers to HIV care.

**Methods:**

Qualitative interviews (*n* = 26) were conducted with PLWH who had experienced barriers to adherence. Participants were referred to the study by staff from Project Trust, a drop in harm reduction and sexually transmitted infection/HIV clinic. The interviews were conducted between May and November 2021. Interviews were recorded, professionally transcribed, coded, and analyzed qualitatively using the integrated-Promoting Action on Research Implementation in Health Services framework.

**Results:**

We identified 6 main themes regarding the acceptability of receiving a long-acting injection to treat HIV, and the acceptability and feasibility of receiving injections at an alternative care site. Participants specified that they: (1) have a general understanding about their HIV care and the importance of ART adherence, (2) prefer a long-acting injection over a daily pill regimen, (3) expressed concerns about injection safety and efficacy, (4) had specific logistical aspects around the delivery of long-acting injections, including location of injection administration, that they believed would improve their ability to adhere, (5) have confidence that they can become undetectable and then complete the oral lead-in required to begin receiving the injection, and (6) see potential barriers that remain a concern for successful adherence to long-acting injections.

**Conclusion:**

To better treat HIV among people who are living with challenging social determinants of health, interventions that include a long-acting injection in a non-traditional care setting may prove to be a promising treatment option.

## Introduction

Treatment for HIV has historically consisted of daily oral antiretroviral therapy (ART). Adherence to ART by people living with HIV (PLWH) is often impacted by experiences with substance use disorder, housing insecurity, food insecurity, incarceration, mental illness, immigration status, and intimate partner violence [1–4]. Health care system factors such as clinic accessibility, provider flexibility and experiences of HIV stigma are other potential barriers to adherence [3, 5]. While there has been extensive research conducted on ART adherence barriers, few studies have explored the barriers that treatment location may have on medication adherence.

A long-acting injection of extended release cabotegravir and rilpivirine (CAB/RPV) is now a Food and Drug Administration-approved treatment for HIV and may be an important tool to improve adherence among populations with high barriers to engaging with traditional HIV care. Early reports on this new intervention have been focused on improving experiences of HIV treatment for people with high adherence and have been conducted in traditional clinic settings [6, 7]. Recent literature has also developed outlines for optimal CAB/RPV candidates, which included past ART adherence and a demonstrated ability to attend frequent clinic appointments [8, 9]. One study found that providers believed that individuals who were experiencing housing insecurity and individuals who were experiencing substance use would not be considered good candidates for CAB/RPV due to the potential for non-adherence and subsequently resistance [8].

Determining whether administering CAB/RPV at an alternative site outside of a tradition clinic may help individuals overcome adherence barriers such as housing insecurity and substance use disorder and is key to understanding the feasibility of utilizing this medication with high-risk populations. However, CAB/RPV initiation requires that a patient is virologically suppressed before transitioning therapies which requires excellent adherence even before starting [6, 7]. There are also concerns that initiating CAB/RPV in someone who may not return for additional injections could lead to resistance [6, 7]. In an effort to provide equitable access to technological advances among high-risk populations, we studied the feasibility and acceptability of alternative care delivery methods, specifically administering CAB/RPV at sites outside of the traditional HIV clinic [10]. In this study, alternative care delivery model refers to the administration of CAB/RPV at any site outside of the HIV clinic where CAB/RPV is currently offered to eligible patients.

In order to design a pilot intervention to test the feasibility and acceptability of administering CAB/RPV at an alternative care site, we conducted a qualitative formative study to assess individuals’ perspectives. We were interested in their experiences and opinions regarding taking daily pills to treat their HIV, barriers to adherence, whether a long-acting injection would allow them to be more adherent, and their thoughts about receiving that injection outside of the traditional HIV clinic setting. Transitioning to a long-acting injection rather than a daily pill may help individuals remain adherent, but there is little evidence on where such injections can best be administered to increase likelihood of adherence. To add to the evidence on alternative care models for vulnerable populations, this study aimed to assess attitudes among PLWH about the injection, and whether a more accessible alternative care site would increase their likelihood of adherence.

## Methods

### Study setting

The study was conducted at Project Trust, a drop-in HIV/sexually transmitted infection/Hepatitis C testing and harm reduction center affiliated with Boston Medical Center (BMC), New England’s largest safety net hospital. On a monthly basis, Project Trust (PT) provides case management and clinical services to approximately 600 individuals. Services offered in the non-traditional PT setting are broad and range from providing individuals with safe injection kits, referrals to detoxification services and resources to address health care needs including diagnosis and treatment of hepatitis C, HIV and sexually transmitted infections, as well as both urgent and preventative medical care. PT serves both PLWH and persons at high risk of acquiring HIV. Of those individuals who received services at PT during 2021, 97% were actively using substances.

### Study sample and data collection

For this analysis, we conducted qualitative interviews with PLWH who frequent PT or who were disengaged from HIV care at BMC. Participants were referred by PT street outreach staff or by the BMC HIV Clinic outreach team who focus on engaging patients out of care greater than 6 months. Individuals who indicated willingness to participate were referred to the study coordinator, who contacted them to schedule an interview. Inclusion criteria specified that participants were at least 18 years of age, English or Spanish speaking, and have a history of non-adherence.

Between May and November 2021, three members of the study team conducted semi-structured interviews with PLWH who met the study criteria. Participants were interviewed in person either at PT, the BMC HIV Clinic, or a public health community site called the “Engagement Center.” Participants provided verbal consent prior to the interview. All interviews were audio-recorded with participant consent. The median length of interviews was 14.5 min. The Boston University Medical Campus IRB approved all study procedures.

### Data analysis

Interviews were transcribed verbatim by a professional transcription service, reviewed for accuracy, de-identified, and uploaded to a secure server for analysis. We conducted a directed content analysis grounded in the core constructs of the integrated-Promoting Action on Research Implementation in Health Services (i-PARIHS) framework [11]. The four core constructs of the i-PARIHS framework are:

1. *Innovation* the intervention being implemented.
2. *Recipients* individuals who are involved in the implementation.
3. *Context* location of the innovation, including the inner context within the organization where the innovation will be executed, and the outer context, including health policy and the community.
4. *Facilitation* how the innovation is introduced and how challenges that arise are addressed [11].

According to i-PARIHS, implementation is successful when the facilitation process supports activation of the intervention, addresses the key contextual factors and meets the needs of recipients [8]. We used i-PARIHS for our analytic framework in order to better understand potential recipient’s feelings about the innovation, how they felt about the new context for the intervention, and their thoughts regarding how to best facilitate it to achieve success.

To conduct the analysis, four members of the research team initially coded two interviews independently, using a preliminary codebook based on the i-PARIHS constructs, creating sub-codes within each construct as needed. The four then met to review their coding and the newly created codes, resolve coding differences, and refine new codes and definitions. This process was continued with an additional three transcripts to develop the codebook. Once the codebook was finalized, the transcripts were then integrated into an analysis template for further coding. Each interview was coded by two team members to ensure coding consistency. The research team then met, reviewed the analytic template, and identified core themes.

It is important to note that during the time when this study was conducted, the FDA approved an optional oral lead-in to replace the mandatory oral lead-in that participants were required to take after achieving viral suppression and prior to starting their injections. The FDA also approved CAB/RPV to be administered bi-monthly rather than monthly as was previously required.

## Results

### Participant characteristics

Table 1 illustrates characteristics of the 26 study participants. Most participants were male (69%); eleven (42%) were white. Time since HIV diagnosis varied from very recent diagnoses (3 months) to individuals living with HIV up to 30 years.

**Table 1:** Participant characteristics (*N* = 26)

Characteristics	Frequency *N* (%)
*Race/ethnicity*	
White	11 (42)
Black	11 (42)
Latino/a	10 (38)
*Gender*	5 (19)
Male	18 (69)
Female	8 (31)
*Age/years*	
18–34	10 (38)
35–49	10 (38)
50–64	6 (23)
*Education level*	
High school or less	16 (62)
Higher education	9 (35)
Unknown	1 (4)
*Time since HIV diagnosis*	
< 2 years	9 (35)
2–15 years	9 (35)
16–30 years	6 (23)
Unknown	2 (8)

## Themes

We identified 6 main themes regarding the acceptability and feasibility of receiving a long-acting injection to treat HIV and the potential of receiving that injection outside a traditional clinic setting. These themes, along with illustrative quotes from interviews, are described below.

### Participants are knowledgeable about their HIV care and the importance of ART adherence

Participants spoke confidently about their care and what their laboratory values indicated about their health. Participants were motivated by being “undetectable” or working toward being undetectable. Participants understood that they needed to stay on their treatment over the course of their lifetime in order to maintain their health status. One individual stated *“I know that I need to be on this medication to stay alive and have a better life for myself, for my future children and everybody in my life” (#23).* Participants identified their overall health and well-being as a long-term priority and indicated a thorough understanding of their HIV care.

Despite recognizing the importance of ART adherence, participants cited several barriers associated with adhering to daily oral medication. These included forgetting to take their pill, prioritizing substance use, housing insecurity and non-HIV-related health issues as barriers to adherence. When asked about what they disliked about taking a daily oral medication, one participant stated:

*It’s hard because I am currently homeless and in active addiction, and it is hard to get in to see a doctor to even get the prescription. And it’s hard to get it filled. And then, once I [finally] get through all those hoops, to hold on to the medication for [the] entire month is nearly impossible. (#11)*

Participants consistently articulated that while they wanted to participate in treatment, the numerous barriers in place made adherence challenging.

### Participants prefer a long-acting injection over a daily pill regimen

In most interviews, participants stated that treatment with long-acting injectable ART was preferable and would work well with their lifestyles. Participants cited the convenience of receiving a long-acting injection as a motivating aspect. One individual explained:

*…because as long as you make it to the place to get that one month shot, you don't have to worry about anything else until the next month's appointment to get that one shot again. So, it eliminates a lot of pressure, a lot more responsibilities and stuff. So, I'm for it. (#17).*

Participants believed that condensing their treatment into a single dose administered once a month would be less of a burden on their day-to-day life in comparison with taking a pill every day.

Participants also described other benefits of a long-acting injection, such as not needing to carry their daily oral ART on their person and not having to worry about their medication being stolen, as reasons they would prefer the injection compared to the oral medication. One participant shared, *“I don’t have to remember to take it. I don’t have to go through the hoops of picking up the medication. Like I said, I don’t have to be scared that it’s going to be stolen from me on the street by people. Literally everything about it sounds wonderful.” (#13)* Participants spoke of housing insecurity preventing them from having spaces to store their belongings including medications, and many participants stated that they would prefer to not carry any medication on their person, especially their ART.

Participants also believed that an injection would be better for their mental health. One participant stated “*Well, I mean, it wouldn't own you. It wouldn't consume too much time. They [injections] would minimize what diagnosis feels like in your life.” (#1)* Participants explained that their oral ART acted as a daily reminder that they had a lifelong health condition, and many indicated that this had a negative effect on their mental health. Participants felt that the long-acting injection would be less of a reminder, and would benefit their mental health.

### Participants expressed concerns about injection safety and efficacy

Participants raised potential side effects of transitioning to the long-acting injection as a concern. One participant remarked *“… because it's new, I would love to see what happens with other people first, how it works before I become a guinea pig….” (#2).*

Participants were also concerned about the potential long-term effects of injectable ART, stating *“I don't like the idea of a shot usually…anything that's going to be in my body for a month…[that] scares me…” (#11).*

Participants also asked about the effectiveness of the new medication. One participant stated *“I guess what's like the risk factor my body is going to reject the medication and becoming detectable again” (#9)*. Several participants acknowledged that they had worked hard to take their daily ART and were concerned that during the transition to the injectable, their viral load may become detectable. Many participants stated that they would want to talk to a clinician prior to switching regimens to ensure that questions they had about the injections safety and efficacy would be answered.

### Participants had specific logistical aspects around the delivery of long-acting injections, including location of injection administration, that they believed would improve their ability to adhere

Participants emphasized that the location where they received the injection should pose minimal interruption to their daily routine. During interviews conducted at the Engagement Center, one participant stated *“Is there any way if you can do it here [Engagement Center Tent]? That would be the best, the way [this place] is set up. The tent [or the] homeless shelter” (#15).* Participants also reported that it was important that the space they receive their injection be safe and private. One stated *“… I will be willing to receive it anywhere as long as it's a safe environment. [I would need to know] the place is legit. Not like some sketchy back alley or with a mad scientist.” (#5)* While some participants indicated they would prefer to receive care in the HIV clinic over an alternative location, many stated that they would prefer to continue to receive their treatment at the community locations they accessed during their day.

Participants brought up transportation as a potential barrier, especially if they were not in close proximity to the injection location. One participant stated:

*I wouldn’t be concerned about forgetting it so much as I would be more concerned about it on a winter’s day. Could I commute to get to it? How would I actually engage if travel was not possible? (#17)*

Several participants asked whether transportation to their appointments would be provided, and several stated that they would have access to transportation services that would help them commute to receive their injection.

Participants spoke about the importance of frequent reminders and communications from their treatment team in order to remain adherent to the injection schedule. A participant specified:

*I have [a] really bad memory. So, email, texts, call, do whatever you [have] to do to make constant communication. And I would say make sure that the person responds… so you know that they got it. (#8)*

During the recruitment process, several participants asked the PT staff for assistance with accessing a cell phone and stated that they often lose or have their phones stolen, which they acknowledged as a barrier to receiving reminder messages.

Some participants stated that they would prefer to bundle their injection appointment with other services and appointments, such as meeting with their case manager, to reduce the overall burden attending a monthly appointment would have on their lives. *“But I'd have to go down there…where my other services are, I would try to line it up with [my other] monthly appointments…so I can bang out both on one day,”* stated one participant. *(#15)* A frequent question was how long injection appointments would take as participants determined whether they would want to schedule their injection on the same day as other appointments and the impact that would have on their day. Participants stated that flexibility with scheduling their injection appointments was crucial to their ability to adhere, and many specified that instability in their lives would make flexibility with appointment scheduling even more important.

Continuity of existing relationships was a motivating factor for many individuals when considering where they would prefer to receive their injection. One participant stated *“…I have [the nurse at Project Trust’s] work number and so …I can reach out and …say, hey or [reschedule] if something popped up [on the day of my appointment].” (#3)* When asked whether the relationship with their provider was important, another participant explained:

*[I like to] know that the people that are treating me are just good people and they actually care about me and [don’t] just want to give me a shot and get it over with, but are people that I can actually build a relationship with. (#6)*

Participants emphasized that having providers who understood their barriers to care and who understood their lifestyle would be a critical support in transitioning to a long-acting injection.

### Most participants have confidence that they can become undetectable and then complete the oral lead-in required to begin receiving the injection

Despite their adherence challenges, participants overwhelmingly believed they would be able to complete the required 28-day oral lead-in to monitor for side effects successfully because they knew at the end they would be able to access a long-acting treatment that would ultimately be easier for them. One stated *“I would try my best for those 28 days. Just knowing that the outcome would be the once a month shot, that would be really helpful.” (#3)* Participants believed that their desire to transition to a monthly long-acting injection would be a strong motivator for them to strictly adhere to the oral lead-in.

### While most participants thought receiving a long-acting injection at an alternative care location was preferable, some saw potential barriers that they believed would impact their ability to remain adherent to the injections

Participants believed that they would remember to get their long-acting injection as long as someone called or texted them to remind them. However, several participants stated that general instability in their lives, including substance use, may be a factor that would prohibit them from being able to receive the injection monthly. One participant stated:

*The only problem I see with the injection is …because my life isn’t stable right now, I can’t sit here and say yes. Realistically, like, in a month from now, I could be in a detox, I could be in a CSS [Crisis Stabilization Service], I could be in a halfway house, you know what I mean. I can’t give myself the injection…, I can’t pick up a prescription for it. The pill form is at least very flexible. (#1)*

Participants felt that having to present to a care site would be a potential barrier if they were in programs that prevented them from traveling to receive their injection.

## Discussion

In this cohort of PLWH who also experienced housing insecurity, substance use disorder, and other adverse social determinants of health, participants indicated strong interest in long-acting injectable ART and saw potential benefits of receiving it at a location outside of a traditional HIV clinical care site. The barriers to adherence that were cited by participants are consistent with previous literature and include forgetting to take their pills, changes in their daily routine, losing their ART, and substance use [1–4, 12, 13]. Study participants believed that long-acting injectable treatments like CAB/RPV would help to address these barriers by lessening the burden that losing medication, remembering to take their pill and the impact of constantly thinking about their HIV diagnosis when taking their daily ART would have on their lives, which is corroborated by the literature studying long-acting medication [12, 15].

Much of the current literature focuses on housing secure patients. Participants in our study reported a lack of stable housing as a critical barrier that impacts their ability to safely store their medication without HIV disclosure or having their medication stolen. Most participants stated that they believed the long-acting ART would address these concerns. Participants in this study also felt that having the option to receive injections at alternative care sites where they have strong relationships and feel comfortable would be an important addition to standard clinical care.

Participants recognized, however, that long-acting injectable ART would not address all the barriers to ART adherence. Due to the unstable nature of homelessness compounded with substance use, many individuals expressed concern that they would not be able to return for scheduled injections if they were incarcerated, in residential treatment or in a detoxification center. Participants also mentioned that if they were unhoused, they were unable to consistently charge their phones and would be unable to receive appointment reminders which would make it challenging for them to remember when injections were scheduled. In the case of CAB/RPV, inability to adhere to subsequent injections or receiving the injection outside of the window during which it needs to be administered raises the possibility of developing resistance due to the long half-life of cabotegravir, which could result in exposure to only one form of ART [13, 14]. These concerns are consistent with provider perspectives about utilizing CAB/RPV with individuals who experiencing housing insecurity and substance use disorder [8, 9]. Though long-acting injectable therapy has been promising in other fields such as addiction and psychiatry, real-world studies suggest that long-term adherence remains a problem [15, 16]. Thus, in order to realize the promise of long-acting ART for this population, the medication must be delivered in the context of effective multi-pronged outreach efforts which is consistent with current literature [8]. Further, efforts should be made to address the social determinants of ART adherence. Delivering long-acting ART in the context of supportive housing, residential treatment, and other alternative venues are opportunities to achieve viral suppression with oral ART, which is a requirement to receive long-acting medications, and may improve adherence. Additionally, robust evidence supports the co-location of addiction treatment with HIV services to improve long-term ART adherence [17, 18].

Our study also identified several important new insights for clinicians seeking to deliver CAB/RPV to vulnerable patients. Despite the new methods of delivering ART, the ease of the injection itself may not solely improve adherence, and the care delivery site and model are likely to be critical. Participants consistently emphasized the relationship that they had with the care team at the non-traditional site as important for long-term adherence. They identified several specific areas that improve these relationships as compared to relationships with providers in traditional settings including open communication and the ability to directly contact their care team through means that are considered non-traditional, such as texting. Participants also emphasized that they would prefer to receive care in locations and programs where they felt comfortable with the team prior to initiating the long-acting injection.

A factor that was not assessed during this phase of the study, but is important to consider in implementation, is the cost of CAB/RPV. The majority of individuals interviewed during this study qualify for the Massachusetts HIV Drug Assistance Program (HDAP) which helps individual who are HIV positive and below a specific income level pay for their HIV medications [19]. HDAP allows individuals who qualify to receive their HIV medications at no cost to themselves. A full cost analysis to assess the financial impact that administering CAB/RPV outside of the traditional clinic may have on institutions and patients is recommended.

During the period that this study was conducted, the CAB/RPV injection was approved by the FDA for every other month administration, and the oral lead-in was made optional, which offers greater flexibility. Future injectable agents with longer intervals between treatment or that can be delivered to achieve (and not just after) viral suppression would provide more options for patients.

This study has limitations. First, as with all qualitative studies, our findings may not be generalizable beyond the population studied. Our sample was constituted disproportionately of men and white people. Although our findings may not be generalizable to other populations, locations, or time periods, eliciting perspectives of this population is crucial to designing potentially feasible interventions and is likely transferable to other settings trying to find methods to engage high-risk populations into life-saving care. Second, this study may be subject to social desirability and selection bias as participants were aware that these interviews were formative work for a future study to test the feasibility of implementing CAB/RPV in alternative care sites. Yet, it is critical to understand their perspectives in order to design useful interventions to improve their engagement and retention in care, since this population of individuals who struggle with adverse social determinants of health and barriers to health care engagement is often under-represented in research. Moreover, this raises an issue of cultural competency in the delivery of new therapeutic regimens that must continue to be addressed as a part of the “Ending the HIV epidemic” toolkit.

## Conclusion

The United States CDC’s HIV campaign, Ending the HIV Epidemic (EHE), cites 4 pillars as critical to ending the epidemic: “Diagnose, Treat, Prevent, and Respond” [20]. Implementing a strategy to provide long-acting injectable ART to vulnerable populations at alternative care sites addresses the “Treatment” and “Response” pillars in a manner that, to the community affected, may help to ensure increased access to improved individual care, as well as contributing to improved public health. PLWH who experience homelessness and high rates of substance use are knowledgeable and motivated to receive ART but face multiple barriers to daily adherence. Delivering long-acting injectable ART at sites outside of the traditional clinic setting was generally endorsed by this study’s participants as a means to improve ART adherence and promote care engagement, despite persistent barriers including substance use and housing instability. The perspectives and experiences of PLWH must be included as interventions to deliver long-acting ART to vulnerable populations are designed and implemented.

## Data Availability

All data generated and used in this research was independently collected by the study team. Data in this manuscript will not be available publicly in order to maintain all individuals privacy and per requirement by the study institutions IRB.

## Abbreviations

ART: Anti-retroviral therapy
BMC: Boston Medical Center
CAB/RPV: Cabenuva and rilpivirine extended release injection
EHE: Ending the HIV Epidemic
i-PARIHS: Integrated Promoting Action on Research Implementation in Health Services
PT: Project Trust
PLWH: People living with HIV
